# Comparing approaches for selection, development, and deployment of extended reality (XR) teaching applications: A case study at The University of Newcastle Australia

**DOI:** 10.1007/s10639-022-11364-2

**Published:** 2022-10-20

**Authors:** Murielle G. Kluge, Steven Maltby, Caroline Kuhne, Darrell J. R. Evans, Frederick Rohan Walker

**Affiliations:** 1 Centre for Advanced Training Systems, The University of Newcastle, Medical Sciences Building Rm 317, Callaghan, NSW 2308 Australia; 2 School of Biomedical Sciences & Pharmacy, Faculty of Health & Medicine, The University of Newcastle, Callaghan, NSW 2308 Australia; 3 School of Medicine and Public Health, Faculty of Health and Medicine, The University of Newcastle, Callaghan, NSW 2308 Australia; 4 Faculty of Medicine, Nursing and Health Sciences, Monash University, Clayton, VIC Australia

**Keywords:** Extended-reality, Virtual-reality, Higher education, Technology implementation

## Abstract

The use of extended reality (XR) technology in education offers many advantages for transferring knowledge and practical skills training at the higher education level. As a result, many Universities over the past 5 + years have undertaken pilot programs to both develop XR content and assess how to best implement it within existing teaching and learning systems. Unfortunately, very few of these efforts have included structured evaluation or documentation. As such, limited published evidence exists to inform processes and approaches that may assist or hinder broad scale implementation. This leads many Universities to unnecessarily commit significant time and resources to testing identical or similar approaches, resulting in repeated identification of the same or similar challenges. In response to this situation, The University of Newcastle, Australia decided to systematically document the approach for selection, development and implementation of four new virtual-reality (VR) teaching applications. The current paper contains a detailed intrinsic case study, outlining the process and critical elements that shaped the selection of suitable teaching content, software development, hardware solutions and implementation. Details are provided on how decisions were made, what components were considered helpful, challenges identified, and important lessons outlined. These findings will be useful to organisations and individuals as they look to develop pathways and processes to integrate XR technology, particularly within their existing training and educational frameworks.

**Supplementary Information:**

The online version contains supplementary material available at 10.1007/s10639-022-11364-2.

## Introduction

Universities and educational institutions are exploring the use of extended reality (XR) technologies as teaching resources, including virtual reality (VR), augmented reality (AR), virtual learning environments (VLEs) and 360-degree video content. XR encompasses technologies which incorporate real- and/or virtual-combined immersive environments, and includes VR and AR. VR refers to fully-digital environments, where the user is fully immersed in a virtual environment via a head-mounted display (HMD) and interacts with virtual elements, typically via hand-held controllers or hand motions (Slater & Sanchez-Vives, 2016). By contrast, AR overlays virtual objects onto a view of the real-world environment where users interact via mobile devices or using AR lenses (e.g. Pokemon GO, Google Glass) (Azuma et al., 2001). 360-degree video refers to spherical recordings that are filmed with a view in every direction simultaneously, which can be displayed in immersive headsets or on 2D screens. We note that in earlier literature, the term VR was also applied to include VLEs, where the virtual environment is viewed on a 2D screen. In some instances, distinctions were made for VR delivered via an HMD using the term immersive VR (iVR) (Merchant et al., 2014). In the current manuscript, we use “VR” to exclusively refer to HMD-based delivery and “VLE” to applications displayed on a 2D screen. XR technologies are increasingly being applied in many areas, including education and training, with technology industry members and educators advocating for further uptake (Hollander, 2018; Pomerantz, 2019; Wang et al., 2018).

In universities, educators are developing their own XR applications, typically driven by motivated individuals, departments, or specific funding initiatives which are tested as pilot applications. Diverse disciplines are generating VR and AR teaching applications in a range of areas, particularly in engineering and health/medicine (Bouaicha et al., 2020; Falah et al., 2015; Garfjeld Roberts et al., 2017; Hernandez-de-Menendez et al., 2020; Hood et al., 2021; Nicholson et al., 2006; Vieira et al., 2017). Similarly, some companies are providing subscription services of standardised VR and AR software applications for undergraduate education and other training.

Overall, existing applications largely fall into three core domains. First, the modelling of complex structures, concepts, or phenomena allowing the trainee to manipulate scale, viewpoints, rotation, and time (e.g., architecture and modelling). Second, the delivery of procedural skills training within a safe and low consequence setting (e.g., surgical procedures and skills). The third, visualisation of a realistic environment or scenario which cannot be readily accessed in the real world (e.g., underwater or space).

There is emerging evidence assessing the relative benefits of XR technology in teaching. The best-documented benefit of XR in this context is increased student motivation and engagement (Garzón et al., 2020; Jensen & Konradsen, 2018; Mikropoulos et al., 1998). XR allows the interaction with manipulation and visualisation of virtual components (e.g. scale, rotation, viewpoints) as well as the visualisation of complex concepts using different perspectives and approaches. In these areas procedural training using XR is particularly beneficial for the development of practical skills (Bouaicha et al., 2020; Canto et al., 2013; Falah et al., 2015; Garfjeld Roberts et al., 2017; Kluge et al., 2021; Legault et al., 2019; Nicholson et al., 2006; Vieira et al., 2017).

As many studies highlight, the use of XR technology alone does not necessarily improve learning outcomes, and the integration of XR into the teaching space is complex (Huang et al., 2019; Mulders et al., 2020; Southgate et al., 2019). Effective use of an innovative educational training solution depends on multiple factors, including the connection to curriculum design, learning outcomes and the students’ needs, the willingness and ability of teaching staff to implement the solution, as well as the ease of technology integration into existing teaching context (Buchner & Hofmann, 2022; Proserpio & Gioia, 2007; Rogers, 2003; Seufert et al., 2021; Straub, 2009). These intricacies associated with the use and uptake of a new technology and teaching modality are likely contributing to the fact that XR technology as a sustainably integrated teaching resource remains in its relative infancy. Despite the growing number of individual VR and AR software applications, very few are currently used in tertiary education as established, functional and scalable training resources across an organisation (Radianti et al., 2020). In their comprehensive systematic review on the use of VR in higher education Radianti et al. (2020) outline the growing number of articles on VR applications, their domains and content areas, whilst highlighting limited evidence for their integrated utility in teaching. Recently Marks and Thomas (2021) describe the practical implementation of a purpose-built VR/AR laboratory in an Australian university. Their study is one of very few outlining the real-world cost and requirements of deploying XR technology in higher education, highlighting the need for more work investigating XR technology infrastructure and classroom integration (Marks & Thomas, 2021). To this point, current research and development activities surrounding the use of XR training software commonly occur as one-off pilot studies and focus on comparing training outcomes to alternative teaching modalities rather than their functional integration into the curriculum. There are practical barriers associated with the delivery of any new technology which cannot be assessed during pilot testing alone. Very little research has focussed on documenting the strategies and approaches used to 1) identify suitable teaching content for XR development, 2) develop or source software or 3) ensure effective classroom integration, ongoing management, and the sustainment of the teaching application.

Key gaps also include the holistic evaluation of real-world costs for development and implementation, hardware options and infrastructure requirements and time impost on teaching staff across XR technologies.

This case study systematically reports data and relevant insights on content selection, software development, delivery modality, maintenance, and distribution strategies for four newly developed XR teaching applications at The University of Newcastle. The intent was to provide a holistic description of relevant considerations, decisions, issues and solutions identified throughout the process.

Specific objectives include the provision of insights into:

- the processes used to manage and select suitable teaching content from across the university
- costs, teaching staff time impost, and infrastructure demands associated with software development and classroom integration for each XR application
- staff and student feedback on the process and final teaching tool
- and a comparison of different approaches and solutions for software selection and implementation

Whilst XR technology appears to have great potential to change the educational and training landscape, few examples and objective evaluations of how XR technology can be structurally integrated into an existing framework are available within the literature. As such the insights from this case study may inform and guide educators and institutions on their own pathway towards adoption of XR technology.

Outcomes from this case study suggest a benefit of facilitating technology integration from an executive level by providing clearly defined guidelines from the outset, specifying approval pathways for suitable content, hardware distribution and infrastructure support. Benefit was also identified by providing developers and educators with subject matter expertise to support the process of XR adoption, development, and implementation.

By exposing the specific issues, complexities and key requirements that were faced and addressed at the University of Newcastle this case study provides suggestions and data to support the expansion of XR technology at-scale and potentially improve teaching and learning outcomes. While this case study focussed specifically on VR technology, the lessons learned may also be relevant to the integration of any innovative teaching technology in a university context.

## Methods

### Study conception and design

**Fig. 1:**
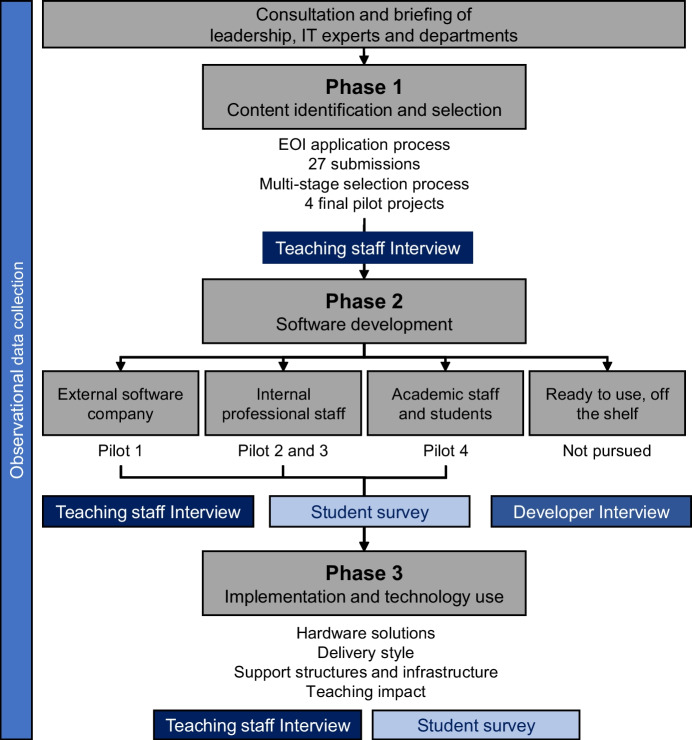
Case study design and data collection process. This case study collected, and documented data throughout the entire process of XR integration into existing courses. Data was evaluated across 3 distinct phases: 1) the selection of suitable teaching content, 2) the development of software and 3) the implementation and use of the XR solutions within their teaching courses, including the evaluation of teaching outcomes. Measures (depicted in shades of blue) included observational data throughout (e.g. costs, time), teaching staff and developer interviews and student surveys at targeted stages, at the time points indicated. EOI = expression of interest

The current intrinsic case study was proposed as part of a new educational design framework to promote teaching innovation at The University of Newcastle. The research group intended to conduct a systematic cost–benefit analysis using a mixed-methods design to capture real world data on the potential strategies for development and integration of XR technologies into the University’s teaching curriculum. The intent was to identify practical avenues and barriers for the use of XR at the university and to inform future pathways and processes for technology integration. The *digital simulation technology evaluation pilot 1* (STEP1) was launched in 2018, with funding support from the Deputy Vice-Chancellor Academic’s office. The case study was structured into three phases (Fig. 1).

#### Phase 1: Content identification and selection

The activities and approaches used to identify and select suitable teaching objectives and goals to be translate into a XR tool, as well as the desire and willingness to integrate technology amongst teaching staff. Extensive discussions with key academic units (Colleges/Faculties, Schools and IT department) on the outset of the project guided and informed the project selection process resulting in a multi-stage expression of interest application process. All interested teaching staff were required to identify teaching objectives and obtain approval from their academic line manager.

#### Phase 2: Software development

This phase focuses on the different approaches used to either create or obtain XR software or content. Specifically, focused on the comparison of bespoke software development by an in-house software development team, an external software company, sourcing of readily available, off-the-shelf software, and the creation of content directly by teaching staff and/or students.

#### Phase 3: Implementation

This phase describes the methods used to integrate the developed teaching applications into their respective courses and teaching environments. It includes the relevant hardware solutions, support structures required for implementation as well as feedback from staff and students on the teaching impact.

The overarching approach and each project phase was informed and guided by extensive scoping discussions with departments heads, IT experts as well as interview sessions conducted with teaching innovators already using XR technologies at the University of Newcastle (Kluge et al., 2022). Four pilot applications received funding, technical, and project management support for the creation of one XR teaching application each (termed Pilot 1–4 in the current manuscript). Of note, project management support was provided by an appointed project manager, who was a member of the research team also involved in the collection of observational data throughout. All pilot projects and the developed teaching applications were subjected to detailed data capture with the intent to compare differing approaches for software development, implementation strategies, and collect teaching staff and student user feedback.

This case study focuses on reporting the processes and solutions relating to XR technology implementation rather than the evaluation of individual training tools themselves. The content of individual teaching applications, both the software solution and the specific teaching objectives, were considered during the decision-making process throughout. However, these aspects were not central to the objectives of the current study and are beyond the scope of this manuscript. Detailed descriptions of the teaching tools were omitted to ensure anonymity of the teaching staff and study participants.

### Setting

Observational data collection commenced in August 2018 with the launch of the *digital simulation technology evaluation pilot 1* (STEP1) and concluded in December 2020 with the end of semester.

The expression of interest application, identifying suitable pilot studies, was distributed and open from the 1^st^ of February to the 4^th^ of March 2019, which was followed by an extended multi-stage selection process. The announcement of final four STEP1 pilot projects was made on the 1^st^ of August 2019. Participant recruitment occurred after the selection of the final four STEP1 pilot projects in October 2019. Whilst the implementation phase was originally planned to occur in semester 1 and/or semester 2 of 2020, the development of some pilot applications was delayed into 2020 due to limited in-house software developer capacity re. As a result, interviews with staff and developers, as well as student surveys occurred at different time points throughout 2019 and 2020 as each individual project completed relevant phases (Pre- development phase, pre- implementation phase, and final feedback). Only Pilots 1 and 3 were integrated into their respective curriculum in semester 1 (March) and semester 2 (September) 2020, due to unanticipated effects of the COVID-19 pandemic / restrictions.

### Participants

Participants in this study were recruited from the teaching staff at The University of Newcastle, Australia who were directly involved in the STEP1 pilot projects and students enrolled in the courses utilising the developed XR tools. Developers working on the XR software creation were also recruited from either the professional staff at the University or by contacting the development company. All teaching staff and developers involved were approached individually and invited to participate in the formal data collection via one or multiple interview sessions. It was made clear that participation in interviews was separate to and not a requirement to receive funding or project support. Written, informed consent was obtained from all participants prior to commencement of the interview.

Students with exposure to the pilot applications in their courses were recruited through their connected online learning platform. Participation in both online surveys was voluntary, on an ‘opt-in’ basis and informed consent was given by ticking a box and continuing to the survey. The pre-survey was distributed via the existing course learning platform within the relevant course material for one week before exposure and closed on the day of exposure to the XR application. The post-survey appeared on the learning management platform the day following exposure to the XR tool and was open for one week.

### Measures and data collection

This case study incorporates comprehensive data collection of observational data, survey and interview data surrounding the selection process, software creation, hardware solutions and integration strategies of four new XR teaching applications developed as independent pilot projects (Fig. 1).

#### Observational data

Quantitative data, including costs, timeframes, number of applications, and meeting schedules were collected and documented by the project manager, who was a member of the research team. Observational data collected during meetings and as the pilot projects progressed were collected by two or more research members independently. Key elements were discussed and summarised across all observers.

#### Self-report data

Questions for the self-report interviews and surveys were constructed using an iterative methodology following a three-step process: 1) Defining constructs and content domains, 2) item generation, and 3) determining the format. Final questions can be found in Supplementary methods 1. All self-report constructs (interviews and surveys) aimed to capture barriers, issues, and unexpected challenges.

Three formal, semi-structured interviews were conducted with teaching staff, using an open-ended question script. Interview questions were drafted based on the priority areas and constructs focusing on a) existing experience, motivation, expectations, and beliefs and b) feedback on and satisfaction with each project phase. Interview questions also addressed domains related to the perceived impact and benefit of the developed teaching application. Interview sessions were conducted prior to the development of the VR tool, post-development but prior to implementation, and post-implementation after utilisation of the application in the classroom. This approach sought to capture changing attitudes, expectations, and experiences throughout the process. A single semi-structured interview was developed for software developers which sought to place the project process and outcome (VR application and co-development with teaching staff) in context with other technology projects. Due to COVID-19 restrictions and meeting schedules, interview questions were transcribed into a fillable PDF format and distributed to developers via email to fill out at their own time.

Student online survey questions used a 5-point Likert scale format and aimed to capture students’ expectations, general attitudes and feedback on the VR tool and its training impact.

The pre-, post- exposure surveys were distributed using SurveyMonkey (SurveyMonkey Inc., San Mateo, California, USA, www.surveymonkey.com).

### Study size

In total ten teaching staff, 43 students and two software development teams participated in this study. Three research members were actively involved in the collection of observational data.

Pilot 1) Twelve (12/43) students responded to the online survey and both teaching staff participated in all three interview sessions separately. The software development team filled out the interview as a fillable PDF document as a group of two designers, one coder and the company’s project lead.

Pilot 2) The VR application was not integrated into the teaching course due to COVID-19 restrictions, the single teaching staff member involved in the development was interviewed across all three interview sessions. One software developer filled out the PDF version of the interview questions.

Pilot 3) A total of 31 students completed the pre-training survey and 27 completed the post-training survey. A total of eight teaching staff participated across the interview sessions. Staff were interviewed individually, with four participating in pre-development interviews, four in post-development / pre-implementation and four in post-implementation. Participation across the three sessions varied due to staff leaving / joining the University and differing contributions to the respective project phases. Two software developers filled out the PDF version interview as a team.

Pilot 4) The VR application was not finalised in time to be integrated into the teaching course due to numerous delays including COVID-19 restrictions, however the three teaching staff members involved in the scoping and development were interviewed.

### Data analysis

Interviews were audio-recorded and summarised non-verbatim by the interviewer. Digital transcripts were proof-read and approved by interviewees. Transcripts or filled PDF versions were qualitatively assessed by two researchers independently, followed by a discussion relating to nodes using NVIVO Pro 12 software (version 12.4, 2020, QSR). Common themes were manually identified, sorted, and coded by topic area and project phase. These included: experience with XR technology; motivation; expectations and feedback on project phases; value areas, challenges and lessons learned.

### Ethics statement

All research was reviewed and approved by The University of Newcastle Human Research Ethics Committee [Study reference: H-2019–0338].

## Results

### Phase 1: Content identification and selection

#### Expression of Interest application

Applications to the Expression of Interest (EOI) were open to teaching staff from all faculties and schools. This required an educator to submit a short brief of the teaching and learning concept that they would like to have developed, which was submitted for approval to their immediate (School Level) supervisor and subsequent sign-off by the respective Head of School. Once the EOI was endorsed it was submitted to the STEP1 portal by email. A total of 27 expressions of interest were received from all faculties (supplementary table 1). While the application document specifically requested applications targeting a specific learning goal or teaching objective, which would benefit from XR technology, only ten applications focused on a single and specific teaching goal. The remainder (n = 17) provided an existing course outline or a description of the overall teaching objectives within the course. The most common teaching domain targeted by proposals was practical training (n = 14), which was in its current form difficult or impossible to provide. This included training of medical procedures or activities under dangerous conditions.

The need for soft skills development via roleplay and the creation of a virtual patients or clients was requested in 12 submissions, while others focused on complex concept visualisation (n = 9) or immersion into a remote, inaccessible and/or artificial environment (n = 6).

#### Selection process

**Fig. 2:**
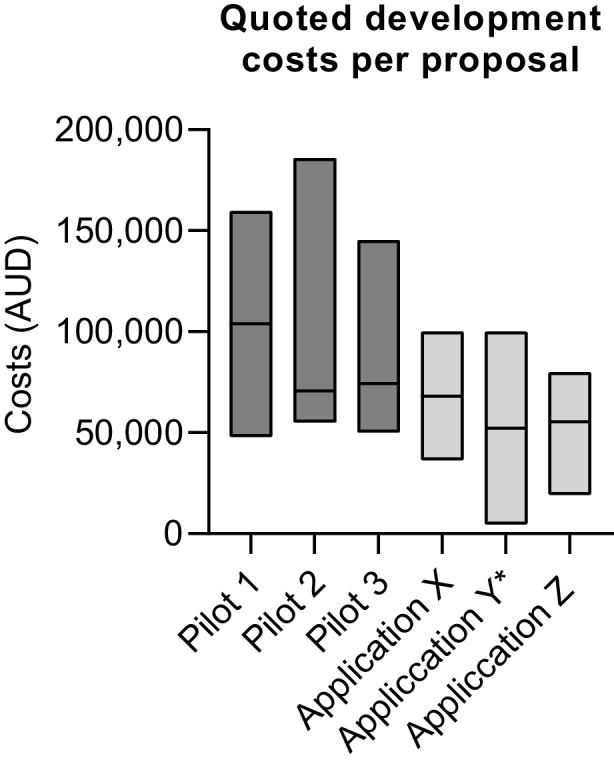
Quoted developmental costs for short-listed XR applications. Data is presented as floating bars (max and min) with line at median. Dark grey bars indicate quote values for the three applications approved for professional development. Light grey bars represent quotes for applications that were subsequently not progressed for development. Numbers of quotes received for each application ranged from 2–4. *quotes received excluded costs associated with required 360 videography

Due to the high proportion of initial applications that contained multiple learning objectives beyond the scope of the current project or technology capability (n = 17 / 27), the research team provided applicants with an opportunity to refine their initial proposal in consultation with the project manager (17-h of staff time). This included clarification of i) XR technology types, capability, utility and limitations, ii) clarification of STEP1 project scope and iii) funding and logistical limitations. Revised applications were reviewed by a multidisciplinary selection committee comprising varying backgrounds (executive leadership, senior teaching academics, technology experts and a research member), to shortlist six suitable pilot projects using a point system. The point system scored each proposal on its value proposition, focusing predominantly on the learning impact and value on student learning. All six shortlisted proposals involved practical or procedural training as the primary learning outcome. Shortlisted proposals were put out to tender and four external software developers responded to the request for quotation. A total of 18 quotes were received for the 6 pilots. Estimated costs ranged dramatically across developers and pilot proposals from $20,000 – $186,000 AUD (Fig. 2). The level of detail included with each quote (e.g. timeline, deliverables, procedures) varied across developers, but was not associated with estimated costs.

The tender process allowed the research team and technology experts to develop and discuss a clear idea for hardware solutions and implementation strategies. The final selection and approval of four pilot projects took therefore into consideration the overall project budget, suitability for evaluation and compatibility with the selected hardware solution (the Oculus Quest virtual reality headset). Approved projects were provided with funding to support software development and implementation. Additionally, each pilot received technology / project management support from a project manager on the STEP1 research team who also conducted comprehensive data capture.

### Phase 2 software development

**Fig. 3:**
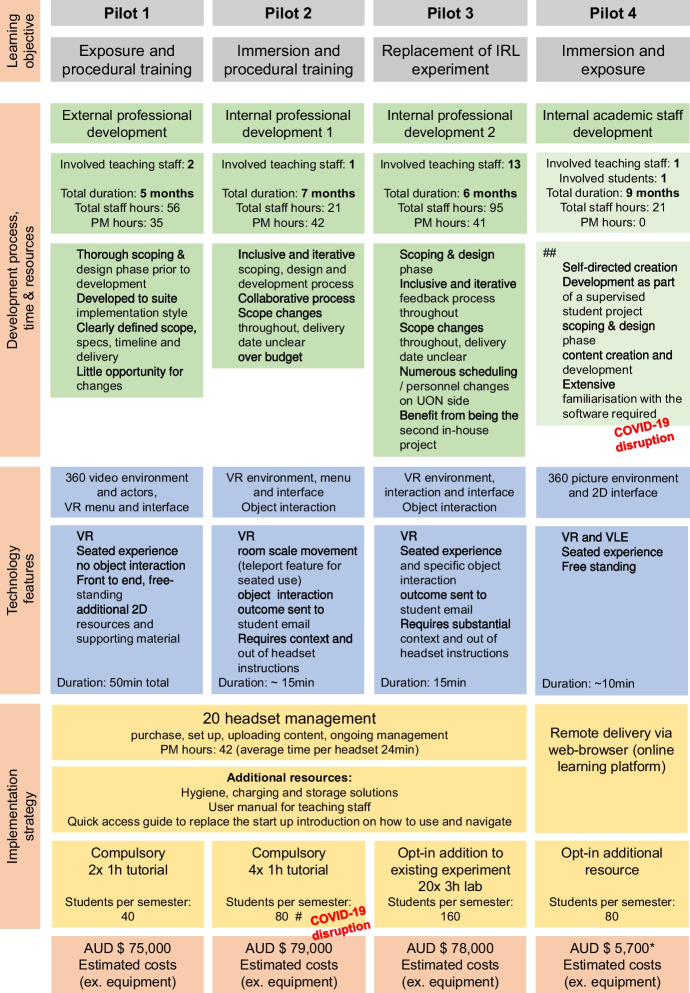
Overview of XR pilot development and implementation. Comparison of the four approved XR applications, including pilot objectives, development, application features, implementation strategy, supporting resources and final costs. ## altered approach from initial pilot proposal, # delayed and not utilised in classroom due to COVID-19, *cost estimates under-represented due to incomplete data capture, CGI = computer-generated imagery; PM = project manager; VR = virtual reality

Based on the submitted applications three different approaches to XR software development were applied and assessed (Fig. 3): i) external development by a commercial development company (Pilot 1), ii) internal development by an in-house professional IT team (Pilot 2 and 3) and iii) self-directed development by academic staff/ educators using a commercially available software program (Pilot 4). None of the submitted applications to the EOI submitted a proposal that could be addressed by a readily available, off-the shelf software solution. In collaboration with the software developers and in alignment with overall case study resources the decision was made to develop all pilot applications for delivery in a HMD virtual-reality headset. Pilot project 1 utilised a blend of CGI interface and 360 video. Pilot applications 2 and 3 developed fully CGI VR environments and Pilot 4 generated a VR application that could be used also as a virtual learning experience on a 2D screen, combining 360 images and 2D interface items by using commercially available software. Pilot application 1 provided exposure to and procedural training on a paediatric patient and represented a training opportunity of a core speech pathology assessment, which previously only occurred on student peers. Pilot application 2 allowed students to explore a crime scene, interact with and connect criminology theories to evidence found within the virtual space. The interactive CGI environment allowed practical training of a skill which had previously only been taught in theory. Pilot application 3 included the visualisation of radioactive principles at different scales and practical measurements of virtual radioactive materials in different environments. The existing lab experiment is cost-intensive and time-consuming, including access to and contact with radioactive materials. Pilot 4 provided students with the opportunity to be oriented within a pharmacy, a training activity not consistently addressed in the classroom.

#### Timeframes, costs and resources

All professionally developed applications received a budget of $ 70,000 AUD for software development, whilst the academic staff-developed application (Pilot 4) costs $ 5,000 AUD (per year) for software licencing fees. The first professional in-house developed pilot exceeded the budget by $ 5,700 AUD due to additional work hours by the developers beyond the initial quote.

All pilot projects commenced in September 2019. The in house developed VR applications were developed sequentially, as the professional staff did not have capacity to develop two applications simultaneously. The total duration of software development by external professional developers was five months (Pilot 1) and seven and six months for the internally developed teaching applications (Pilot 2 & 3, respectively). Pilot 4 had the longest absolute development time of 9 months, by self-directed independent development (but the lowest overall costs). We did note that the content creation timeline was heavily impacted by the emergent of the COVID-19 pandemic, as Pilot 4 teaching staff had competing requirements to transition all existing teaching resources for remote delivery. In contrast, development of Pilot 3 by internal professional staff was minimally impacted (2-week delay), as software developers were able to work remotely and continued meeting via web-conferencing.

In addition to the total time and direct costs of development, we also documented staff time commitments not included in the project budget (Fig. 3, green). Estimated project manager time was comparable across all three professionally developed projects (approximately 5—6 days / 35 – 42 h per pilot). In contrast, estimated staff time varied considerably across projects, which was largely associated with the number of staff involved in each project. Pilot 2 had only one teaching staff member involved, who spent approximately three workdays (21 h) on development. Pilot 1 included two academic staff members, who together logged an estimated 8 days (56 h). Pilot 3 included a total of 13 teaching staff, with varying contributions to the scoping, development, and implementation phases, logging a total of 14 days (98 h) during development. As noted, Pilot 4 development was heavily impacted by COVID-19 restrictions and ultimately was developed by a part-time student as part of their course research project, with supervision from a single teaching staff member. Neither the student nor the teaching staff member documented their time commitments at the time. On reflection, the supervising staff member estimated approximately three days (21 h) of time spent, but no data was available from the student.

#### Comparison of development approaches

Pilot projects 1–3 were developed for deployment in a head-mounted display (HMD), whilst Pilot 4 can be delivered via either HMD or 2D computer screen. For Pilot 4, the 2D modality was primarily used for distribution via an online learning management system, due to COVID-19 restrictions preventing face-to-face teaching.

All professional developers spent considerable time on scoping, to understand and consider the teaching needs, teaching requirements, technology capabilities, and technology restrictions. In-house development applied an agile-based, iterative project management approach, whilst external developers clearly defined and specified the final application a priori, before undertaking content development via a sequential waterfall approach. The iterative approach used by the in-house developers included fortnightly meetings with teaching staff and unscheduled informal feedback throughout. Both in-house-developed pilots (Pilot 2 & 3) underwent multiple scope changes and had unclear timeframes and delivery dates. Pilot 3 benefited from being the second in-house application developed, as developers adjusted their iterative approach to clearly define priorities and required features, streamlined development processes and communications, and re-used VR aspects between pilots (e.g., email reporting integration). In contrast, the external developers conducted an extensive scoping phase and presented multiple delivery options before initiating development, which prompted teaching staff to specify their specific needs and preferences prior to development. Once defined there was little opportunity to adjust content or change scope. Timeframes, logistics, milestones, delivery dates and required resources were clearly defined early in the process, allowed teaching staff to manage their time and plan ahead to provide specific input or resources as required and with minimal delays.

Implementation strategies were considered to varying degrees during the development of each application. The externally developed application (Pilot 1) included extensive early consideration of implementation, which resulted in a free-standing application that contains task and navigational instructions within the VR application itself. Implementation and delivery approach were not specifically considered for the internally developed applications. Pilot 2 required the development of additional instructional materials to be delivered by teaching staff in a tutorial setting outside of the headset. Specific instructions on task objectives were intentionally not integrated into the Pilot 3 application in order to create a free-form setting in which students are encouraged to creatively explore options, and allowing flexibility across different courses.

In Pilot 4 the approach was adjusted due to the COVID-19 pandemic. Scoping and design were predominantly driven by teaching staff independent of the research project manager. Content development was completed by the student.

#### Interview feedback

Developers were questioned on the difficulty and complexity of the developed pilot application. Each pilot was reported to have minor project-specific complexities; however, overall development was rated as “easy” or “very easy” for all three XR applications.

**Fig. 4:**
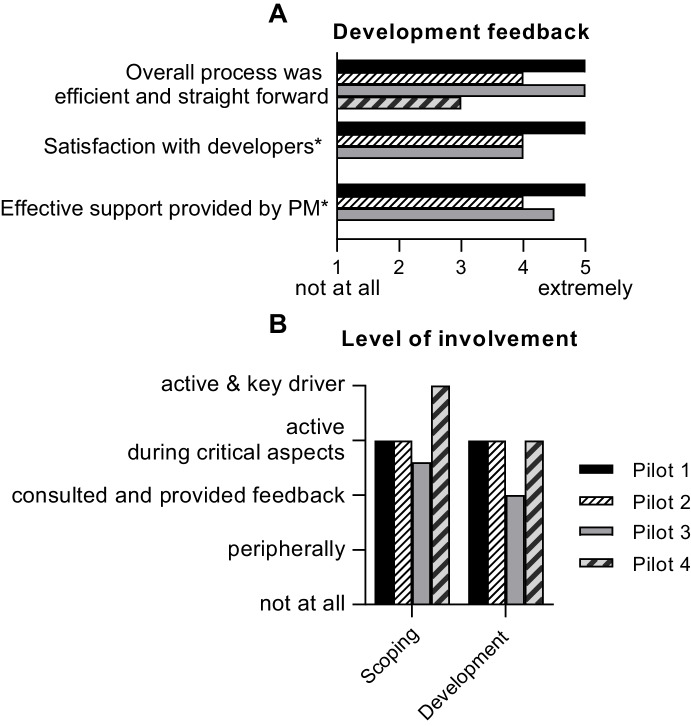
Teaching staff feedback on the development process. Teaching staff feedback on **A**) application development and **B**) their level of involvement for their respective XR teaching application. Numbers of respondents = pilot 1, n = 2; pilot 2, n = 1; pilot 3, n = 6; pilot 4, n = 1. *N/A for pilot 4, PM = project manager

Despite different developmental approaches applied, no differences were observed between pilot projects in terms of teaching staff feedback. All teaching staff who collaboratively developed their tool with software developers were very satisfied with the overall process, including interaction and communication with the developers and the effectiveness of project manager support (Fig. 4A). Teaching staff who worked with the external development company (Pilot 1) were particularly pleased to have their voices included as the audio in the final VR application. All teaching staff stated that they had a high level of input throughout development and were satisfied with their level of involvement (Fig. 4B).

Consistently positive feedback was provided by teaching staff in an open-ended question on the development process:

*“We very much liked the developers who provided us with thorough information that was not too far out of our technical depth from start to finish. A very personal and efficient service.” Pilot 1*

*“Overall happy with most of the process.” Pilot 2*

*“It went very smoothly, regular meetings and feedback kept everyone up to date and helped find small issues that could be resolved.” Pilot 3*

Similar positive feedback was provided by the professional developers:

*“Some questions were not answered due to commercial confidence but overall, it was a great working relationship.” Pilot 1*

*“The SME’s [subject matter expert = teaching staff] could have come to see the application a bit more during development, but despite this development went smoothly.” Pilot 2*

*“We spent a decent amount of time at the beginning, exploring different directions for the project. This meant that by the time we started actually developing the application we were pretty confident about what we were doing and what the priorities of the different features were. As a result, we didn't waste much time with changing direction mid-production.” Pilot 3*

It is relevant to note that Pilots 1 and 2 were led by their respective course coordinators (who were also the original applicants on the expression of interest). These project leads were involved in VR development and implementation and took responsibility across the entire process. For these pilots, the working relationship between developers and teaching staff members was considered productive and focused by both sides. In contrast, pilot projects 3 and 4 had more than five applicants on their initial expression of interest, which included teaching staff, course coordinators, heads of school and teaching advisors. Further, team members changed during development, with staff departing and joining the university (Pilot 3) and interfering obligations due to COVID-19 leading to adjustments (Pilot 4). This meant that there was not a consistent lead across all project phases for these applications. For Pilot 3, this resulted in repeated and extended discussions on the specific learning goals and subject matter to include in the application. When queried about the working relationship with teaching staff members for this pilot, the developers stated *“…there were many SMEs [subject matter experts] and at times it was hard to get clear decisions out of them. […] If we were to do the project again, it would be beneficial to make sure from the beginning that we had fewer SMEs and ensuring that they are the right ones.”* Of note, this pilot also had the highest commitment in terms of teaching staff hours during development.

Academic staff who self-directed development of their XR application (Pilot 4) indicated they were very happy with the opportunity to develop their own tool. However, they indicated that initial familiarisation with the software / technology was very time-consuming. They stated that filming of content took longer than anticipated. Although technical and general support was available from both the research project manager and the software company, the educators of this pilot did not contact or request assistance during development or implementation. Overall, the communication between the STEP1 research team and the educators for this project was very low compared to the regular contact that was established with other educators. This may be due to the extended timeframe, changing priorities, as well as the nature of the self-directed development approach. Input from the STEP1 project manager may have appeared less valuable. Staff stated that they felt poorly supported throughout the developmental process.

*“The provided online tutorials were not very helpful. It would have been better had there been some tech support from the company. There was no guide on how to use the software and it was clear that it was not made for academic staff with little tech experience.”*

Teaching staff and software developers were “very satisfied” with their final developed VR applications (n = 12/12). All teaching staff stated that the tool addressed all or the main proposed teaching goals (n = 9/9). Further they all had confidence in its potential effectiveness (n = 9 stated it very likely for the VR tool to be an effective and useful teaching tool).

### Phase 3: Implementation

#### Technology set-up, management, and deployment

The research team and project manager consulted with internal and external technology experts to select hardware options, in parallel with the selection and development process. Considerations included the overall budget, technology requirements and flexibility to support all 4 pilot tools. At the time (June / July 2019), the Oculus Quest headset was chosen, as it was considered the most cost-effective, untethered, stand-alone, and user-friendly HMD. Further, the company launched their enterprise software management solution (Oculus for Business), which supports remote management of multiple headsets simultaneously and provides customisation options on the deployment of VR applications (e.g. kiosk mode, blocked administrators functions, etc.). To compare the logistics of individual device versus bulk device management strategies, the headsets were used in their consumer-grade setup in semester 1 2020, then transitioned to the enterprise-enabled mode in semester 2. Comparative data was collected for the headset set-up, loading of VR applications, and ongoing headset maintenance (summarised in Table 1).

**Table 1:** Comparison of headset management between consumer-grade and enterprise solution

	Consumer grade Oculus Quest	Oculus for Business Oculus Quest for Business
Costs	$ 700 ea./ 14,000 AUD total	$ 1,550 ea. / 31,000 AUD total + $ 255 ea. annual licence fee
Initial set up	• Headset and account install via phone application (single headset at a time) • Individual 10 min ea. / 3.3 h total	• Headset install via phone application and enterprise account (up to 25 headsets simultaneously) • IT support for system setup on PC • Grouped 30 min total
Loading of content	• Requires PC with external software, driver and change of settings for sideloading • Some initial IT experience required • Individual uploads 10 min ea. / 3.3 h total	• Requires PC with account and server storage • Remote and grouped application installation • individual resource load (videos/pictures) requires external side-loading software and driver
Ongoing management	• By individual headset	• Remote and grouped • When updates / device status not confirmed requires individual headset checks
Headset experience	• Access to all Oculus apps and features • User navigation through menus required to launch teaching content	• Control over content deployment & display and safely features • Kiosk-mode supports direct entry into teaching application without user navigation

A fleet of 20 headsets were purchased for approximately $31,000 AUD total (including hardware costs, shipping and customs fees), which were managed by the STEP1 research team and made available to teaching staff for implementation. Additional implementation costs included consumables and products to support the roll-out of four VR teaching applications to up to 300 students (totalling $5,500 AUD), encompassing storage and transport solutions, hygiene masks and covers, cleaning products and a UV-light clean box.

Setup, content loading and hardware checks for a new consumer grade headset required 20 min per device at initial setup (approximately 6.5 h for all 20 headsets). Whilst the initial installation of all required programmes, drivers, accounts, and changes of headset settings required some technical expertise, ongoing upload and management of content was relatively straight-forward and quick (3 min per headset). A major disadvantage of using the consumer-grade headsets in a teaching setting was the inability to control content delivery or restrict access to specific applications. Conversely, enterprise-enabled headsets allowed educators to restrict application access and directly load only the teaching application via a “kiosk mode”. Teaching staff who used both the consumer-grade and enterprise versions commented “*It [the delivery] felt so much easier this time [using the enterprise version]. It worked well for the app to be there when the students turned on the headset*.” Although the enterprise software provided the ability to remotely upload and manage application deployment, not all content files could be deployed via the enterprise portal. None-embedded video files had to be loaded to the headsets manual as the upload was not supported. Seamless integration requires consideration of the enterprise approach during application development and/or technical expertise to manually load additional content onto each individual headset. The initial setup of the enterprise management accounts and connection to the university IT infrastructure required considerable technical expertise and direct input and approval from the university’s IT department. All HMD-deployed teaching applications required supporting documents and materials including a general user-manual and a one-page quick access guide, which were developed by the research project manager.

#### Implementation strategies

Three of four VR teaching applications were successfully integrated into their teaching courses in 2020. Pilot 1 was implemented as a compulsory task in two hour-long tutorial sessions, with 43 students having exposure to the final tool across two sessions. Students using the Pilot 1 VR tool spent an average of 50 min in the headset. Staff and students were “satisfied” or “very satisfied” with the implementation strategy (100%), rated the navigation and use of the tool as “easy” or “very easy” (100%) and reported only two minor technical problems. Whilst students stated that exposure time was mostly adequate (n = 9; only 2 stated they would have liked more time), teaching staff would have liked to allocate more time for a general introduction of the technology and headset features. They noted that device familiarisation took longer than anticipated, exceeding the allocated 10 min in the tutorial setting. Of note, the enterprise solution with kiosk mode resolved this issue.

Pilot tool 3 was integrated into an existing laboratory setting as an optional resource to complement the existing lab experiment (Fig. 3). Approximately 160 students used the VR teaching tool between August and November 2020. On average, students indicated they used the VR application for 19 min (range = 5 to 90 min) and 77% (n = 17) indicated the amount of time was adequate (n = 5 would have liked more time). Most students (n = 19/22) were “satisfied” or “very satisfied” with how training was integrated into the course. The majority (77%; n = 14/18) reported that VR use was “easy” or “very easy”, with only one student indicating it was “difficult”.

Interviewed staff, exposed to Pilot application 3, noted that the technology was easy to setup and use and that students engaged with the tool as they had hoped / anticipated (n = 4), but that they would include additional guidance in future roll-out. They noted some practical difficulties with using the technology, but that this only affected a minority of students. When queried on why they did not use the VR tool, two students indicated that VR made them sick / dizzy, two students did not want to put on the headset, and one student preferred to complete the real-world experiment only. Eight student respondents indicated technical issues, which related to implementation (e.g. low headset / controller charge; n = 4) or application design (e.g. instructions, user interactions, email export functionality). In open answer responses, students highlighted the need for an orientation / explanation of the training, for example:

*“There was not a whole lot of explanation given about how to use the virtual environment and it didn't really seem as if the instructor presenting this information had much experience with it/knew how to use it very well.”*

*“Instructions presented were not clear.”*

*“Needed more explanation. Didn't really expand on something we could just do in class.”*

Pilot application 4 was made available to students via the web-based online learning management system as an additional resource (via a learning management system). Due to the relative freedom of the teaching staff to self-develop their teaching application and impacts of COVID-19, Pilot application 4 was implemented without the research project manager or research team. Participation in the current case study was voluntary and not mandatory for funding support. As a result, no data was collected on the implementation phase for this tool.

While Pilot 2 was finalised in February 2020, it was developed for implementation in face-to-face tutorial sessions (four hour-long tutorials with 20 students each) and the course was transitioned to a fully remote format due to COVID-19 pandemic restrictions. Despite the teaching staff being eager to implement the application, its integration is currently on hold until on-campus tutorials reconvene.

#### Teaching impact and overall tool feedback

##### Pilot 1 feedback

**Fig. 5:**
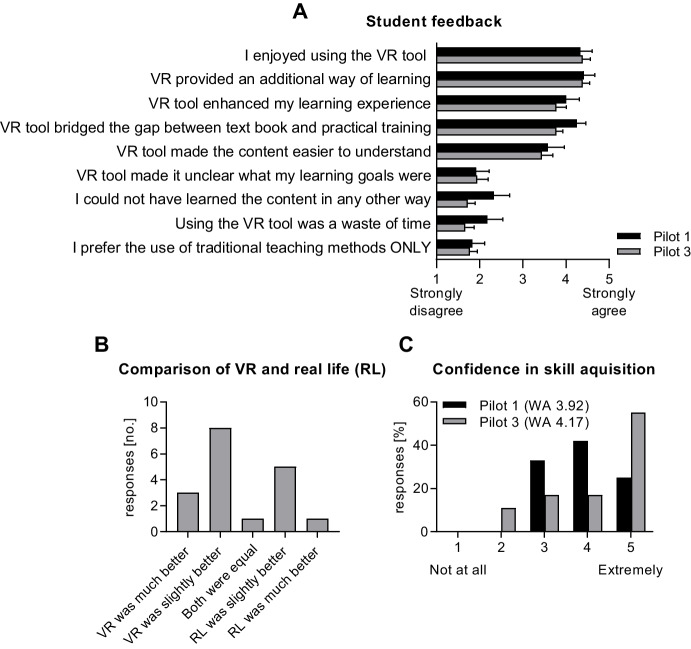
Post-exposure student feedback. **A**) General student feedback following VR application implementation, for teaching applications 1 and 3, presented as weighted averages (WA) ± SEM. **B**) Student feedback directly comparing real life (RL) laboratory experiment to the VR tool for teaching application 3. **C**) Rates of confidence in skill acquisition after VR training exposure for Pilot 1 and 3. Number of responses: Pilot 1, n = 12; Pilot 3, n = 18

Teaching staff were extremely satisfied with the developed tool, stating that it was extremely effective and useful in addressing the intended learning objective and had a positive impact on students learning. Students agreed that the tool was enjoyable (weighted average 4.3, 1 = strongly disagree, 5 = strongly agree) and added to and enhanced their learning experience (weighted averages 4.4 and 4.0; Fig. 5A). In open answer format questions, staff and students commented:

*“It was a fantastic substitute for actually conducting [the procedure] during our placements.” Student*

*“I think this was a wonderful way of introducing us to [the procedure] and really did feel like a live experience.” Student*

*“The main positive impact was around being given the opportunity to practice a skill they would otherwise not have had.” Staff*

All surveyed students reported at least some confidence that they learned the content delivered by the VR tool (“somewhat” n = 5, “very” n = 5, “extremely” n = 3) and 100% indicated knowing how to perform the specific procedure after VR training. In comparison, before completing VR training, only 44% (n = 7) stated that they knew how to perform the procedure.

Teaching staff stated that the VR tool added value by increasing student engagement, providing consistent content exposure, and supporting practical skills training. No disadvantage was indicated relating to the use of VR in their course and within their subject matter area. Teaching staff perceived the VR tool to be a replacement of an existing teaching modality, highlighting that the content was previously not taught effectively. In contrast, students indicated that the content could have been taught using other methods but noted that VR was highly suitable in bridging the gap between textbook and practical training.

One student indicated that the application made them feel sick and would have preferred the content to be delivered using traditional methods. However, both staff and students expressed a desire for the continued usage of the VR tool as well as an expansion of further content, increased complexity, and increased difficulty of the virtual scenario (n = 5).

### Pilot 3 feedback

Teaching staff were “extremely satisfied” with the created teaching tool and indicated it was “extremely useful” in addressing learning outcomes but noted that a structured assessment documenting any impacts on student learning is needed. Most students (13/18) were confident that they had learned the content delivered via the VR tool. Students agreed with the statement that they “enjoyed using the VR teaching tool as part of the course” (mean = 4.4; 1 = strongly disagree, 5 = strongly agree) and that the “tool provided an additional way of learning” (mean = 4.4). Students particularly liked and commented on the efficiency of the VR tool compared to the real-world experiments, due to a “time warp” function that allowed them to complete measurements rapidly. Student quotes included:

“*It was an interesting and unexpected way to conduct part of the lab. It felt much more engaging than if it was just shown in a video or demonstrated by an instructor.”*

*“It was a really cool way of learning and allowed for some fun playing around and easy data collection.”*

Teaching staff stated that they believed the VR teaching tool to be better than the existing teaching approaches, and that the VR tool could replace or be used in addition to the existing approach. When asked to directly compare the VR teaching tool to the real-world experiment, 11/18 student respondents reported that VR was “slightly” or “much better” than the real-world experiment, while 6/18 preferred the real-world experiment (n = 1 ranked both approaches as equal). The majority (n = 12/18) would prefer to have the VR tool used in addition to the real-world experiment or available as optional education content (n = 5), while none felt it should replace the real-world lab and one felt the VR tool should not be included at all. All teaching staff indicated they planned to continue using the tool in the future.

## Discussion

In this case study, four VR teaching applications were developed as independent pilot projects, three of which have been successfully utilised within their intended teaching courses at The University of Newcastle, Australia. All four pilot projects and their created applications were subjected to a comprehensive data capture from start to finish, including their selection, development, implementation, and end-user feedback. This was done to gain comparative data and assess cost–benefit as it related to different approaches for the use of VR technology in tertiary education. In line with previous research, it was found that there is merit in using VR applications to increase student engagement and learning (Jensen & Konradsen, 2018). Beyond this, the results identified the resources and key processes that may support, from an institutional and teaching staff perspective, the effective utility and implementation of XR technology as a scalable teaching resource. It is further reassuring that the core findings are consistent with and expand the results from a recent study at the University of Sydney, evaluating the use of an established a virtual-and augmented-reality laboratory (Marks & Thomas, 2021).

### Organisational level endorsement and support

While experimentation with innovation is exciting, and readily captures the attention of new audiences, the reality for large institutions is that the broad scale adoption of ‘cutting edge’ technologies are problematic from both a cost and risk perspective. Academic institutions, while often having many staff, tend to have relatively limited capacity to adopt new processes. This is in part because most staff are already committed to existing modes of delivery for teaching and learning. Further, changes to teaching and learning infrastructure can have unanticipated negative impacts on the delivery of teaching and learning objectives. Effective implementation of new teaching approaches, XR or other, therefore requires the exploration and development of implementation strategies, internal organisational endorsement, and support structures, in addition to the creation of new software and procurement of XR hardware. Important considerations involve how the new ‘enabler’ will be implemented, what staff training will be required, who will be involved in project management, what hardware and software will be required, what service agreements and licensing fees will be involved, how technology will be nested within key organisational units (e.g. disciplines, schools, faculties, colleges) and how oversight will occur (e.g. approvals, procurement, coordination).

Engagement and endorsement with leadership is an important avenue for teaching staff to ensure ongoing infrastructure and funding support throughout the lifespan of a developed pilot application. Sustainment of XR technology use is a critical gap as recognised in a recent study, which showed that the use of innovative XR prototypes tends to be discontinued after initial evaluation, due to lack of funding and/or technical support for hardware and software updates, despite being considered highly useful teaching tools (Kluge et al., 2022).

One of the priorities of the STEP1 project and the research approach was therefore to engage and involve leadership and managers throughout the University to promote organisational level buy-in and increase the likelihood of future support for effective teaching applications. Of note, the STEP1 project was made possible through endorsement and funding support from the Deputy Vice Chancellors office (Academic), which funded the software development and hardware and opened the door for the research team to engage with and brief staff and executives throughout the University. This also demonstrated the direction of the university to engage with new teaching technologies and provide confidence and encouragement to educators to contribute.

Active engagement and involvement with leadership and department heads was ensured by utilising a two-step authorisation pathway for the EOI process. Staff were required to obtain clear approval and endorsement from their academic line manager and department heads to apply to the STEP1 program, ensuring leadership were aware and briefed on the intent to create an innovative teaching tool within their department.

The selection committee involved in the shortlisting and selection of the STEP pilot projects was similarly comprised of executive leadership, members of the IT department, teaching supervisors and academics. This level of open communication and active inclusion resulted in the universities IT department taking a keen interest in the project. IT offered in-kind contributions to support in-house software development as well as practical IT support to assist with hardware integration onto the universities Wi-Fi network.

While both University leadership and IT were actively engaged and endorsed the pilot applications on the outset and during development, it is important to note that none of the developed VR applications have received ongoing financial or structural support since completion of the initial STEP1 project funding. Two factors may have contributed to this lack of continuity. In 2021 the university underwent a staffing restructure, resulting in a turnover of leadership and managers. Secondly, this may be, in part, due to the dramatic shift in teaching demands which occurred in response to the COVID-19 pandemic. Teaching departments were forced to direct resources towards solutions that would allow remote delivery of teaching content. As all STEP1 teaching applications were designed for in-classroom face-to-face delivery they have received little ongoing attention or support despite being successfully utilised by educators throughout the pandemic. Without institutional level endorsement to support sustainment, the future use of all 4 developed applications is currently unclear. However, as the education sector recovers from COVID-19 restrictions the interest and exploration of XR technology in education is likely to increase again.

### The value of high-level oversight

This case study utilised a structured and process-driven project management approach across all project phases from the EOI application to the implementation of teaching applications. Initially, project management was intended to primarily ensure the collection of relevant data to inform a cost–benefit analysis and support staff. It quickly became apparent that the involvement of the project manager and research team members represented a major (if not the most important) factor for the success of the overall project and for each individual pilot project by substantially driving and guiding the decision processes throughout. All research team members involved in the project have experience in the use of XR technology in teaching and training. Importantly the discussions with leadership and the IT department at the outset and throughout the STEP1 project resulted in the combination of highly specific insights into the technology and training/ teaching requirements coupled with an organisational-level oversight and knowledge of institutional barriers. This allowed the research team, via the project manager, to draw on and bring into focus considerations from multiple viewpoints. This skillset was particularly beneficial during the selection and development phase of pilot projects, by bringing together leadership views, infrastructure requirements/limitations and practical teaching objectives. For example, an advanced level of oversight was required to identify the need to specify the hardware solution and implementation strategy prior to the selection of the final pilot projects in order to effectively distribute the available resources.

Further, higher-level oversight ensured execution of the EOI and selection processes that were well-defined, transparent, and easy to navigate, which were mutually beneficial to the applicants and the selection committee. The simple streamlined application form, together with the substantial additional support provided to teaching staff by the project manager ensured that high quality proposals were put forward, which could be easily compared against the selection criteria. Providing clarification and assistance during the EOI phase required considerable time and resources from the project manager, however, was a valuable expense as it meant many teaching staff were able to purposefully connect technology to their teaching goals, refining their understanding which would support them in exploring other avenues in the future. Whilst the research team was not involved in the actual selection of projects, they provided valuable insights and clarifications to the selection committee as they had spoken directly to all teaching staff that were shortlisted. The research team provided valuable insights into each application, and general oversight of scalability, implementation and sustainment options required to make an impactful decision on suitable pilot projects.

Specific and general experience with XR technology in an education context was also highly beneficial in supporting communication between teaching staff and software developers. Whilst educators found it difficult to understand and imagine the proposals and suggestions put forward by the software developers, software developers found it difficult to connect to the underlying pedagogies, teaching goals and objectives. Having an experienced translator present to guide and manage the communication was thus of benefit. Finally, the insights from the project manager were used to develop user manuals and protocols for students and staff to use in the classroom.

In summary, the following elements were found to have been particularly beneficial to the overall success of this case study as they underpinned decisions made throughout:

1. Expectation management and transparency throughout all project phases
2. Organisational-level involvement and engagement
3. Clear understanding of existing IT and infrastructure barriers and limitations
4. Experience with XR technology, specifically its use in a teaching context
5. Clearly defined processes, criteria, and guidelines
6. Pre-defined implementation strategy and identified hardware to guide selection and development of content
7. Provision of technical, subject matter and management support for teaching staff, including project oversight

These elements were largely provided, meaningfully connected, and managed by the project manager who provided direction and guidance to the teaching staff throughout the STEP1 case study.

### Clarity around requirements, limitations, and logistics

During the EOI process, the decision was made to identify the technology type, distribution, and delivery method prior to the selection of suitable teaching content. This choice was made for two reasons. Firstly, this approach allowed the effective distribution of available resources across multiple courses and STEP1 pilot projects by using the same hardware for all projects. Secondly, it was identified that the delivery system itself should be considered during the design and content choices of the application. XR technology has the capacity, in principle, to be used across a large range of use cases and dimensions, both in subject matter, duration and interactivity. As a result of this flexibility, applications can vary greatly in their requirements (e.g. obstacle free space, navigational instructions, familiarisation time, battery life and recharge time etc.). Logistical and practical restrictions such as room sizes, space, student numbers, staff availability, type of hardware, charging intervals and duration, safety constraints and content life- cycle need to be identified and considered during the development phase. This ensures that the application runtimes, degree of freedom, level of interaction, internal and external instructions are suitable to local resources and constraints.

Within the pre-determined the delivery solution, the implementation strategy of each pilot application was dictated by the educators and the practical logistics of their course structure, course delivery and student numbers. Pilot 1 used a highly structured and compulsory implementation strategy whilst pilot 3 was delivered using an opt-in approach in parallel to existing lab activities. Whilst all staff and students reported their particular approach to be effective, the VR tool in pilot 1 was given a clear place within the curriculum. As a result, the pilot generated a clear pathway for ongoing use and has consistently been used since completion of the study. For pilot application 3 continued use has been less consistent, as educators have indicated lack of time to update the course structure to integrate the. Successful ongoing implementation of XR will likely depend on the application being integrated into the standard curriculum and course delivery.

A similar approach of pre-determining the delivery restrictions was used at the University of Sydney (Marks & Thomas, 2021). Their implementation strategy was postulated and clearly defined by the establishment of a dedicated technology room first, before encouraging staff to develop software which would then be delivered within the existing facilities. The establishment of a XR-dedicated room appears to be an effective delivery modality, however, is associated with larger upfront cost associated with logistical planning, room re-fitting, technical support as well as operation and management cost. In this case study, a hardware booking system approach was applied which allowed staff to borrow headsets for the required training times with delivery of XR training in their standard classroom / laboratory settings. This type of delivery was made possible using untethered and mobile HMDs that do not require the use and connection to computers or tracking stations.

The specific implementation strategies (i.e. either a dedicated room or mobile booking system), should both be effective provided the solution is supported by and integrated into local IT infrastructure to ensure ongoing support and maintenance. Management of software, hardware, updates and charging of 20 consumer-grade headsets within this case study was time-consuming, a point that was also raised for the equipment used in the dedicated technology laboratory (Marks & Thomas, 2021). Further, whilst the user-friendly and mobile consumer-grade HMDs used in this case study can be obtained at a price point of $700 AUD per headset, they are unlikely to be supported within the Universities IT infrastructure due to security concerns and account management issues. We therefore recommend investment in an enterprise software management system, as it allows control over content delivery, secure access, remote device management and update management to streamline maintenance.

It is worth noting that there is an expanding number of organisations developing XR content for university education, beyond the activities formally reported in traditional published academic literature (e.g. ("ClassVR"; "EON Reality"; "Liminal VR"; "MYRACLE.IO")). The number of professionally and commercially available software applications are fast growing and so does the market for packages that support the delivery of a comprehensive solutions which include hard-, software and sustainment. Based on the issues and barriers highlighted in this case study, an external XR provider may be an attractive solution for universities. However, this approach representing a large commitment whilst also accompanied by uncertainty and risks as the technology and market is still developing rapidly. Specifically, the longevity of any hard- or software solution is unclear at the time of purchase. This issue can be seen in the recent history of XR technologies, where stand-alone consumer headsets first emerged in the last ~ 5-years and there have already been several cycles of hardware upgrades and renewal during this time phase (e.g. Oculus Go to Oculus Quest 1 to Oculus Quest 2). Additionally, enterprise software management solutions are only just emerging now with one of the central platforms already being discontinued after only 2 years in use. Whilst solutions relating to software compatibility across platforms are currently being developed the selection of an external provider restricts universities to a specific hardware device and consequently the type and number of applications that can be used. It is often not clear how long the provider will be able to support any device and what additional software and applications the institution may be required to access in the future and whether they will be supported within that solution.

### Focus on learning objectives and priority areas

The importance of connecting the use of digital technologies in teaching to a specific teaching goal and objective has been widely reported (Makransky & Petersen, 2021; Mulders et al., 2020; Radianti et al., 2020). Further, it is imperative to consider how the content can transition purposefully into a multi-sensory experience without becoming distracted by the technology capabilities (Straub, 2009). With these aspects in mind the STEP1 program focused on how the identified learning goals transitioned into a VR teaching tool.

Pilot project 1, which defined a clear and specific learning goal from the start, resulted in a streamlined development process and a free-standing, easy to integrate application with clear instructions and learning outcomes. In contrast, learning goals were less clearly defined for Pilot project 3 and changed several times, leading to a longer developmental process and a final VR application that received criticism for lacking a clear intent and purpose. Changes in focus were compounded by a change in staffing. In this project, both the teaching staff and developers were excited by the technological capability to create a “free-world” experience that provided students with flexibility but failed to connect this functionality to a specific learning goal. The decision to apply a free-world design impacted on the ability of the VR tool to replace the specific laboratory experiment as was initially proposed. Of note, additional instructional documents and briefings were difficult to implement in practice as they required students to take off the HMD headset. This experience highlights the importance of considering the impact any decisions have on the learning outcomes and implementation logistics.

It is also important to note that XR technology has limitations and may be suited for some teaching and learning objectives more than others. Evidence highlights that XR teaching tools can effectively improve practical skills development (Bouaicha et al., 2020; Canto et al., 2013; Falah et al., 2015; Garfjeld Roberts et al., 2017; Kluge et al., 2021; Legault et al., 2019; Nicholson et al., 2006; Vieira et al., 2017). An XR tool addressing practical skills has the most value when otherwise ethically inappropriate, expensive, and/ or logistically impossible to provide. To identify valuable teaching avenues and reduce the time impost on educators, the EOI phase of this study requested staff to specify a learning objective that would fit within the following four areas aligned with XR value-proposition:

1. Flexible off-site and/or remote delivery of content
2. High-risk and/or high-complexity procedural training
3. Reduced costs compared with current provision of content
4. Cross-degree scalable training

Many initial EOI applications failed to specify a specific learning objective or value area, but instead focused on describing the in-principle technology capabilities. Applicants overestimated the capacity and feasibility of XR technology, had unrealistic expectations and failed to connect them to an achievable learning goal. It became clear that many teaching staff lacked the background experience to realistically link the capabilities of XR technology to their teaching needs. Further, when faced with specific decisions during the scoping and development phases, teaching staff often struggled to visualise how the technology could be used effectively and be practically implemented.

Whilst it is theoretically possible to replace an entire course with XR content, doing so is not necessarily achievable nor desired by either teaching staff or students. In this case study, all four applications were developed as support tools, meaning they did not replace a teaching session but were rather used as an adjunct to traditional in-classroom/laboratory teaching delivery. Both staff and students indicated a strong desire for XR technology to not replace face-to-face teaching and training altogether but viewed it as a valuable adjunct to existing approaches. This sentiment was particularly voices by students in pilot 1, who spent the longest time within the VR tool (50 min).

All professionally developed teaching applications relied heavily on the support, expert recommendations and advice provided by the project manager particularly during project scoping, planning, conceptualisation, and initial implementation of the teaching application. The expert provided guidance and support to contextualise the technology, its capabilities, and restraints and how to realistically utilise and implement the tools within their course structure. Expert input was particularly valuable in identifying the key areas of importance throughout the case study. Specifically, the need to i) specify and consider the implementation and hardware solutions during scoping and development, ii) the importance of repeatedly connecting decisions back to specific learning goals and iii) be realistic and aware of the limitations of XR technology use in teaching.

Taken together, organisations may benefit from developing clear guidelines of what teaching content / areas is suitable for XR development, priority areas and the extent to which the technology can be used within their institution. Successful integration of XR technology into the teaching curriculum is likely to occur when a clearly defined teaching objective motivates its creation and informs decisions made during all phases of selection, scoping, development and implementation. The capacity and learning context in which the technology is intended to be used is specific to and should be clarified by each individual educational area early. Teaching staff can then work within the suggested guidelines to develop effective teaching tools, making long-term sustainability and utility of any XR teaching application more likely.

## Conclusion

This case study provides in-depth insights into the local development of four pilot VR teaching applications at The University of Newcastle. The outcomes highlight that the scalable use of XR technology within any organisation, past individual pilot projects, will require a substantial up-front investment, time commitment and development of collaborative and transparent guidelines and processes at an institutional level. Taken together the following key elements of this approach may include i) a pre-defined framework for hardware distribution and ongoing support, ii) clear guidelines and approval pathways for suitable teaching content, and iii) provision of expert support to advise and guide staff throughout the entire process of XR adoption, development and implementation. These same factors may be broadly relevant to the integration of any new innovative technology into university education.

## Supplementary Information

Below is the link to the electronic supplementary material.

Supplementary file1 (DOCX 136 KB)

## Data Availability

The datasets generated during and/or analysed during the current study are not publicly available due to privacy of the human participants in interviews but are available from the corresponding author on reasonable request.

## References

[CR1] Azuma R, Baillot Y, Behringer R, Feiner S, Julier S, MacIntyre B (2001). Recent advances in augmented reality. IEEE Computer Graphics and Applications.

[CR2] Bouaicha, S., Epprecht, S., Jentzsch, T., Ernstbrunner, L., El Nashar, R., & Rahm, S. (2020). Three days of training with a low-fidelity arthroscopy triangulation simulator box improves task performance in a virtual reality high-fidelity virtual knee arthroscopy simulator. *Knee Surgery, Sports Traumatology, Arthroscopy, 28*(3), 862–868. Retrieved from http://ezproxy.newcastle.edu.au/login?url=http://search.ebscohost.com/login.aspx?direct=true&db=s3h&AN=141881540&site=eds-live 10.1007/s00167-019-05526-y 31079163

[CR3] Buchner J, Hofmann M (2022). The more the better? Comparing two SQD-based learning designs in a teacher training on augmented and virtual reality. International Journal of Educational Technology in Higher Education.

[CR4] Canto S, Jauregi K, van den Bergh H (2013). Integrating cross-cultural interaction through video-communication and virtual worlds in foreign language teaching programs: Is there an added value?. ReCALL.

[CR5] ClassVR. Retrieved from https://www.classvr.com/au/. Accessed 12 Aug 2022

[CR6] EON Reality. Retrieved from https://eonreality.com/. Accessed 12 Aug 2022

[CR7] Falah, J., Charissis, V., Khan, S., Chan, W., Alfalah, S. F. M., & Harrison, D. K. (2015). *Development and evaluation of virtual reality medical training system for anatomy education*, Cham.

[CR8] Garfjeld Roberts P, Guyver P, Baldwin M, Akhtar K, Alvand A, Price AJ, Rees JL (2017). Validation of the updated ArthroS simulator: Face and construct validity of a passive haptic virtual reality simulator with novel performance metrics. Knee Surgery, Sports Traumatology, Arthroscopy.

[CR9] Garzón, J., Acevedo, J., Pavón, J., & Baldiris, S. (2020). Promoting eco-agritourism using an augmented reality-based educational resource: A case study of aquaponics. *Interactive Learning Environments,* 1–15. 10.1080/10494820.2020.1712429

[CR10] Hernandez-de-Menendez M, Escobar Díaz C, Morales-Menendez R (2020). Technologies for the future of learning: State of the art. International Journal on Interactive Design and Manufacturing (IJIDeM).

[CR11] Hollander, R. (2018). VR giants are moving into education *Business Insider*. Retrieved from https://www.businessinsider.com/google-oculus-vr-education-2018-8?r=AU&IR=T

[CR12] Hood, R. J., Maltby, S., Keynes, A., Kluge, M. G., Nalivaiko, E., Ryan, A., … Walker, F. R. (2021). Development and pilot implementation of TACTICS VR: A virtual reality-based stroke management workflow training application and training framework. *Front Neurol, 12*, 665808. 10.3389/fneur.2021.665808 10.3389/fneur.2021.665808 PMC8631764 34858305

[CR13] Huang, T.-C., Chen, M.-Y., & Hsu, W.-P. (2019). Do learning styles matter? Motivating learners in an augmented geopark. *Journal of Educational Technology & Society, 22*(1), 70–81. Retrieved from https://www.jstor.org/stable/26558829

[CR14] Jensen L, Konradsen F (2018). A review of the use of virtual reality head-mounted displays in education and training. Education and Information Technologies.

[CR15] Kluge MG, Maltby S, Keynes A, Nalivaiko E, Evans DJR, Walker FR (2022). Current state and general perceptions of the use of extended reality (XR) technology at the University of Newcastle: Interviews and surveys from staff and students. SAGE Open.

[CR16] Kluge MG, Maltby S, Walker N, Bennett N, Aidman E, Nalivaiko E, Walker FR (2021). Development of a modular stress management platform (Performance Edge VR) and a pilot efficacy trial of a bio-feedback enhanced training module for controlled breathing. PLoS ONE.

[CR17] Legault, J., Zhao, J., Chi, Y.-A., Chen, W., Klippel, A., & Li, P. (2019). Immersive virtual reality as an effective tool for second language vocabulary learning. *Languages, 4*(1), 13. Retrieved from https://www.mdpi.com/2226-471X/4/1/13

[CR18] Liminal VR. Retrieved from https://liminalvr.com/. Accessed 12 Aug 2022

[CR19] Makransky G, Petersen GB (2021). The Cognitive Affective Model of Immersive Learning (CAMIL): A theoretical research-based model of learning in immersive virtual reality. Educational Psychology Review.

[CR20] Marks B, Thomas J (2021). Adoption of virtual reality technology in higher education: An evaluation of five teaching semesters in a purpose-designed laboratory. Education and Information Technologies.

[CR21] Merchant Z, Goetz ET, Cifuentes L, Keeney-Kennicutt W, Davis TJ (2014). Effectiveness of virtual reality-based instruction on students' learning outcomes in K-12 and higher education: A meta-analysis. Computers & Education.

[CR22] Mikropoulos TA, Chalkidis A, Katsikis A, Emvalotis A (1998). Students' attitudes towards educational virtual environments. Education and Information Technologies.

[CR23] Mulders M, Buchner J, Kerres M (2020). A framework for the use of immersive virtual reality in learning environments. International Journal of Emerging Technologies in Learning (iJET).

[CR24] MYRACLE.IO. Retrieved from https://www.myracle.io/. Accessed 12 Aug 2022

[CR25] Nicholson DT, Chalk C, Funnell WRJ, Daniel SJ (2006). Can virtual reality improve anatomy education? A randomised controlled study of a computer-generated three-dimensional anatomical ear model. Medical Education.

[CR26] Pomerantz, J. (2019). XR for teaching and learning: Year 2 of the EDUCAUSE/HP campus of the future project. *ECAR research report*. Retrieved from https://library.educause.edu/-/media/files/library/2019/10/2019hpxr.pdf?la=en&hash=306474918AA2F101DDDCABD59E4366AD7244D572

[CR27] Proserpio, L., & Gioia, D. A. (2007). Teaching the virtual generation. *Academy of Management Learning & Education, 6*(1), 69–80. Retrieved from www.jstor.org/stable/40214517

[CR28] Radianti J, Majchrzak TA, Fromm J, Wohlgenannt I (2020). A systematic review of immersive virtual reality applications for higher education: Design elements, lessons learned, and research agenda. Computers & Education.

[CR29] Rogers EM (2003). *Diffusion of Innovations*.

[CR30] Seufert S, Guggemos J, Sailer M (2021). Technology-related knowledge, skills, and attitudes of pre- and in-service teachers: The current situation and emerging trends. Computers in Human Behavior.

[CR31] Slater M, Sanchez-Vives MV (2016). Enhancing our lives with immersive virtual reality. Frontiers in Robotics and A.

[CR32] Southgate, E., Smith, S. P., Cividino, C., Saxby, S., Kilham, J., Eather, G., … Bergin, C. (2019). Embedding immersive virtual reality in classrooms: Ethical, organisational and educational lessons in bridging research and practice. *International Journal of Child-Computer Interaction, 19*, 19–29. 10.1016/j.ijcci.2018.10.002

[CR33] Straub ET (2009). Understanding technology adoption: Theory and future directions for informal learning. Review of Educational Research.

[CR34] Vieira CB, Seshadri V, Oliveira RAR, Reinhardt P, Calazans PMP, Vieira Filho JB (2017). Applying virtual reality model to green ironmaking industry and education: ‘a case study of charcoal mini-blast furnace plant’. Mineral Processing and Extractive Metallurgy.

[CR35] Wang P, Wu P, Wang J, Chi H-L, Wang X (2018). A Critical review of the use of virtual reality in construction engineering education and training. International Journal of Environmental Research and Public Health.

