# Supplementary material for: Comparing approaches for selection, development, and deployment of extended reality (XR) teaching applications: A case study at The University of Newcastle Australia
Source: Educ Inf Technol (Dordr). 2022 Oct 20;28(4):4531–62. doi: 10.1007/s10639-022-11364-2 (PMC9584278; doi:10.1007/s10639-022-11364-2)
Supplement: Supplementary file 1 — Supplementary file1 (DOCX 136 KB) [file 10639_2022_11364_MOESM1_ESM.docx]

**Supplementary Information**

# Title:

# Comparing approaches for selection, development, and deployment of extended reality (XR) teaching applications: a case study at The University of Newcastle Australia

Murielle G. Kluge^1,2#^, Steven Maltby^1,2#^, Caroline Kuhne^1,2^, Darrell J. R. Evans^3,4^ & Frederick R. Walker^1,2*^

*^1^Centre for Advanced Training Systems. ^2^School of Biomedical Sciences & Pharmacy, Faculty of Health & Medicine, The University of Newcastle, Callaghan NSW 2308, Australia, ^3^School of Medicine and Public Health, Faculty of Health and Medicine, The University of Newcastle, Callaghan NSW 2308, Australia, ^4^Faculty of Medicine, Nursing and Health Sciences, Monash University, Clayton, Victoria, Australia.*

**^#^Contributed equally as first authors**

***Corresponding Author:** Prof. F. Rohan Walker, Centre for Advanced Training Systems, The University of Newcastle, Medical Sciences Building Rm 317, Callaghan NSW 2308, Australia. P: 02 4921 5012; E: [rohan.walker@newcastle.edu.au](mailto:rohan.walker@newcastle.edu.au)

Journal: Education and Information Technologies

**Supplementary table 1:** **Overview of STEP1 expressions of interest applications**

Summary of all applications received to the STEP1 EOI process. Data includes proposed courses, topic area and teaching goal / learning goals. Assessment outcomes for each application are listed.

| **Course** | **Topic Area** | **Teaching Domain** | **Outcome** |
| --- | --- | --- | --- |
| 3^rd^ year Bachelor of Chemical Engineering | Membrane reactors and micro-chemical plant design | Simulation of complex structure / concept  Practical training | Suitable |
| 3^rd^ year Midwifery, 4^th^ year Medicine | Obstetric emergency | Soft skills development /  Exposure / training | *Out of scope* |
| 4^th^ year Bachelor of Pharmacy | Administration of injections and immunisations | Practical training | Suitable |
| 3^rd^ year Physiotherapy | Paediatric physiotherapy | Soft skills development  Practical training | Elements suitable  *Out of scope overall* |
| 2^nd^ year Bachelor of Business | Trade logistics | Procedural training  Simulation of complex structure / concept | Suitable |
| 1^st^ and 2^nd^ year B. Science, B. Engineering, B Medical Radiation Science, B. Environmental Science | Radiation processes | Practical training  Visualisation | **Developed** |
| 3^rd^ year Bachelor of Science | Climate and catastrophes | Practical and procedural training | Suitable |
| 2^nd^ year Bachelor of Speech pathology | Paediatric oral muscular assessment | Procedural training / exposure | **Developed** |
| Bachelor of Arts | World Religion | Immersion / Exposure | Suitable |
| 2^nd^ year Bachelor of Pharmacy | Pharmacy | Soft skills development  Orientation | **Developed** |
| Bachelor of Podiatry | Biomechanics | Simulation of complex structure / concept  Procedural training  Visualisation | Suitable components  *Out of scope overall* |
| 1^st^ year Bachelor of Social Sciences | Crime scene simulation | Practical training / exposure | **Developed** (initial proposal out of scope) |
| 1^st^ year Bachelor of Global Indigenous Studies | Cultural awareness | Exposure  Orientation | Suitable |
| 2^nd^ year Bachelor of Law | Physical and fault elements of crime | Practical / contextual skill training | Suitable |
| 2^nd^ year Medical Radiation Science | Radiography | Soft skills development | *Beyond technology capacity*  Quotes obtained |
| 3^rd^ year Bachelor of Podiatry | Podiatry nail surgery | Practical training | Suitable |
| 2^nd^ year Bachelor of Design (architecture) | Architecture | Technology exposure  Practical training | Suitable |
| Bachelor of Nursing, Medicine and Medical sciences | Patient care | Soft skill development | *Beyond technology capacity* |
| Bachelor of Medical Sciences and Medicine | Clinical reasoning | Soft skills development  Procedural and practical training | *Beyond technology capacity* */ out of scope* |
| Bachelor of Environmental Science, Science | Marine world | Exposure | Suitable (Practical logistical difficulties)  Quotes obtained |
| Visual Communications and Creative Industries | Creation of a transdisciplinary creative space | Immersion  Practical training  Technology exposure | *Beyond technology capacity* */ out of scope* |
| 4^th^ year Bachelor of Nutrition and Dietetics | Medical nutrition therapy | Soft skills development | *Beyond technology capacity* |
| 4^th^ year Bachelor of Engineering | Chemical plant design and operation | Simulation of complex structure | Suitable (out of scope) |
| Honours in Nutrition and Dietetics | Hospital orientation | Exposure  Procedural training | Suitable |
| 2^nd^ year Bachelor of Science | Mock interview / job readiness | Soft skills development | *Beyond technology capacity* */ out of scope* |
| 4^th^ year Chemical Engineering | Dynamic process simulation | Simulation of complex structure | Suitable |

**Supplementary Methods 1:**

**Self-report Items content:**

1. Staff Pre-Development Questions (Phase 1)
2. Staff Post-Development Questions (Phase 2)
3. Staff Post-Implementation Questions (Phase 3)
4. Developer Questions (Phase 2)
5. Student pre-exposure/implementation survey (Phase 2)
6. Student post-exposure/implementation survey (Phase 3)

## **Staff Pre-Development Questions (Phase 1)**

**Section 1) Participant information**

- 1. What School do you belong to?
  2. How long have you been employed at the University of Newcastle?
  3. How many semesters of teaching/lecturing experience do you have within the university sector/ how many at UON?
  4. How many semesters have you been managing a course?

**Section 2) Exposure to XR technologies prior to STEP1 application.**

- 1. Did you have experience with XR technologies, for example virtual reality, either through personal or professional experience prior to application to STEP1? Yes/No. If yes, please specify which type:
  2. Have you used any other alternative medium or technology (including XR) to assist with your teaching course? Would you consider yourself to be a person open to new and innovative approaches to teaching? Yes/No. If yes, please specify:
  3. How confident are you with the setup, use and implementation of XR technology in a teaching context?

|  |  |  |  |  |
| --- | --- | --- | --- | --- |
| Extremely confident | very confident | Somewhat confident | not very confident | not at all confident |

**Section 3) STEP1 EOI application**

- 1. How did you become aware of the STEP1 EOI program?
  2. What were your reasons for applying to STEP1?

3.3 What university course and setting will the XR teaching tool be implemented in?

- 1. How many students are enrolled in this course currently?
  2. Are you the coordinator of this course? Yes/No. If no, please specify who is:
  3. What is the teaching goal/ learning objective/ desired outcome of the teaching tool?
  4. What category of teaching goal does your XR tool address?
- Knowledge transfer
- Skill acquisition
- Repetitive practical training
- Assessment
  1. Did you have a preferred technology type in mind when applying for STEP1?

Yes/ No. If, Yes please specify:

- 1. How long do you think the XR teaching content should be?

**Section 4) Development of teaching tool**

- 1. How clearly defined is your image of the final teaching tool in regard to the specific functionality, look and exercises?

|  |  |  |  |  |
| --- | --- | --- | --- | --- |
| Extremely defined idea of how it will function, look and the type of exercises | very defined | Somewhat defined | not very defined | no clear idea of what it would look like or the type of exercises that should be included |

- 1. How willing are you to deviate from the ideas that you have about your teaching tool?

|  |  |  |  |  |
| --- | --- | --- | --- | --- |
| Extremely willing | very willing | Somewhat willing | not very willing | Not at all willing |

Where are you willing to make compromises, where are you not:

- 1. How familiar are you with the process of XR content development?

|  |  |  |  |  |
| --- | --- | --- | --- | --- |
| Extremely familiar | very familiar | Somewhat familiar | Very broad idea | I have no idea how the process might work |

- 1. How much of your time do you think will be involved in the development and creation of the teaching tool?

Do you consider this a major time commitment? Yes/No. Please comment:

- 1. Do you have any concerns about the developmental process of the teaching tool? Yes/No. If yes, please specify:

**Section 5) Implementation**

- 1. Do you have a preference on how the tool should be integrated into the course/ made accessible to your students? Yes/No/somewhat, but nothing specific. Please specify:
  2. If you have a preference for integration, how willing are you to deviate from that?

|  |  |  |  |  |
| --- | --- | --- | --- | --- |
| Extremely willing | very willing | Somewhat willing | not very willing | Not at all willing |

Where are you willing to make compromises? Where are you not:

- 1. Do you have any concerns about the implementation of the teaching tool? Yes/No. If yes, please specify:

**Section 6) General expectations**

- 1. In your opinion how likely is it that the STEP1 project will provide you with an effective and useful teaching tool for your course?

|  |  |  |  |  |
| --- | --- | --- | --- | --- |
| Extremely  likely | very likely | undecided | not very likely | no at all likely |

- 1. What are the expected benefits of this XR teaching tool in your course?
  2. Do you expect any difficulties with the use of XR in your course? Yes/No. If yes, please specify:
  3. Do you have any general concerns about the use of XR technology at the University in general? Yes/No. If yes, please specify:

1. Any final topics and comments you would like to add:

## **Staff Post-Development Questions (Phase 2)**

**Section 1) General satisfaction**

- 1. Overall are you satisfied with the developed XR tool as a final version?

|  |  |  |  |  |
| --- | --- | --- | --- | --- |
| Extremely satisfied | Very satisfied | Somewhat satisfied | Not very satisfied | Not at all satisfied |

If no, please specify what aspects you are not satisfied with:

- 1. In your opinion, how likely is it that the developed XR tool will be an effective and useful teaching tool within your university course?

|  |  |  |  |  |
| --- | --- | --- | --- | --- |
| Extremely likely | Very likely | Undecided | Not very likely | No at all likely |

If you selected unlikely please specify why:

- 1. How well does the developed teaching tool address the teaching goals and objectives you initially wanted to address?

|  |  |  |  |  |
| --- | --- | --- | --- | --- |
| The tool addresses more teaching goals and objectives than originally proposed | The tool addresses all proposed teaching goals and objectives | The tool addresses the main proposed teaching goals and objectives but not all | The tool addresses some teaching goals and objectives but not all | The tool addresses different teaching goals and objectives than those originally proposed |

- 1. Please rate the potential for your developed XR tool to be transformed and utilised outside of your teaching course for any other course or teaching purpose?

|  |  |  |  |  |
| --- | --- | --- | --- | --- |
| High potential for an alternative use case | Some potential for an alternative use case | Might have potential for an alternative use case | Alternative use case would involve substantial investment and modification | No potential for an alternative use case |

If you indicated some potential please specify where it might be useful:

**Section 2) Feedback on developmental process**

- 1. How did the development occur? How was it managed?
  2. How long did the developmental process take (total time start to finish)?
  3. How many University staff members (or students) were involved during the development of this application?
  4. Would you say the developmental process, as a whole, was an efficient and straight forward process?

|  |  |  |  |  |
| --- | --- | --- | --- | --- |
| Extremely efficient and straight forward | Very efficient | Somewhat efficient | Not efficient | Not at all efficient or straight forward |

- 1. Please rate your level of active involvement in the scoping (planning and design phase) of your XR teaching tool.

|  |  |  |  |  |
| --- | --- | --- | --- | --- |
| I was actively involved and a key driver during planning and design | I was actively involved in critical aspects of planning and design | I was consulted and involved in providing feedback and decision making | I was only peripherally involved in planning and design | I was not at all involved in planning and design |

- 1. Please rate your level of active involvement in the developmental phase of your XR teaching tool?

|  |  |  |  |  |
| --- | --- | --- | --- | --- |
| I was actively involved and a key driver during all aspects of the developmental process | I was actively involved in critical aspects of the developmental process | I was consulted and involved in providing feedback and decision making | I was only peripherally involved in the development of the XR tool | I was not at all involved in the developmental process |

- 1. Please rate your satisfaction with your level of involvement during the scoping phase.

|  |  |  |  |  |
| --- | --- | --- | --- | --- |
| I would have liked to be involved more during the scoping phase | I would have liked to be involved more but did not have the time or resources | I am satisfied with the level of my involvement | I would like to have been involved less but can’t see how that would have been possible | I would have liked to be involved less during the scoping  phase |

- 1. What did you like/ not like about the developmental phase and process?
  2. Were there any difficulties you did not anticipate during the developmental phase? Yes/No. If yes, please specify:
  3. Did you feel supported during the developmental process of the teaching tool? Yes/No. If no, please specify:

**Section 3) Implementation**

- 1. How will the tool be used in the future and when? How will students have access to it?
  2. Is this strategy your preferred implementation method or would you prefer a different method? Yes/No. If yes, please specify your preference:
  3. Do you have any concerns about the implementation and use of the XR teaching tool in your course? Yes/No. If yes, please specify:

4. Any final topics and comments you would like to add:

## **Staff Post-Implementation Questions (Phase 3)**

**Section 1) Feedback on Implementation**

- 1. How many students interacted with the technology?
  2. How long, on average, did students engage with the technology?
  3. How was the technology presented/ made accessible to the students?
  4. Were students satisfied with the implementation and presentation of the technology? What did they like/ what did they not like?
  5. Where there students that refused to or were unable to engage/ use the technology?

Yes/No. If yes, please specify:

- 1. What did you like/ not like about this mode of implementation?
  2. Where there any practical issues/ difficulties with this strategy?
  3. Would you like to change the implementation strategy/ or the way the content is delivered in the future? Yes/No. If yes, please specify how:

**Section 2) Student feedback and outcome**

- 1. Did students accept the technology as a mode of learning? Did they use and engage with the XR tool? Yes/No. Specify the level of acceptability:

2.2 How easy was the use of the XR tool?

|  |  |  |  |  |
| --- | --- | --- | --- | --- |
| Extremely easy | easy | Neither easy nor difficult | difficult | Very difficult |

- 1. What was the general student feedback regarding the content and learning outcomes? Did they find useful for learning purposeless?
  2. In your opinion do you think student learning was impacted by the use of the XR teaching tool? Yes/No/ Specify how or why not:
  3. Will you be aiming to continue to use the tool in this course in the future? Yes/No. Specify how or why not:
  4. Is there any reason/barrier that might prevent you from using the tool in the future? Yes/No. If yes, please specify:

**Section 3) General satisfaction**

- 1. On a whole, are you satisfied with the created teaching tool?

|  |  |  |  |  |
| --- | --- | --- | --- | --- |
| Extremely satisfied | very satisfied | Somewhat satisfied | not very satisfied | Not at all satisfied |

- - 1. If you indicated that you are not satisfied with the XR tool please select the reason for this answer:
- Does not address the intended learning objectives adequately
- Insufficient design (look and feel)
- Inappropriate implementation strategy
- Use of the tool is too difficult and/ or time consuming
- Other:

Comment:

3.2 Where does the XR teaching tool sit within the broader context of the course outline?

- XR tool is an addition/ to the current teaching method
- XR tool replaces the previous modality of teaching the content
- XR addresses content that could not be taught previously

3.2.1 If you indicated that the XR teaching tool replaces the previous teaching modality, indicate how it compares to the previous method.

|  |  |  |  |  |
| --- | --- | --- | --- | --- |
| The XR tool is far superior to the previous teaching method | The XR tool is better than the previous method | The XR tool is comparable to the previous method | The XR tool is not as good as the previous method | The previous method is superior to the XR tool |

Please provide a reason for your answer:

3.3 In your opinion, how effective and useful is the XR teaching tool in addressing the learning objectives?

|  |  |  |  |  |
| --- | --- | --- | --- | --- |
| Extremely useful | very useful | undecided | not very useful | no at all useful |

Please provide a reason for your answer:

- 1. Were there any disadvantages in using the XR tool in your class? Yes/no. If yes, please specify the disadvantages:
  2. Were there any advantages in using the XR tool in your class? Yes/No. If yes, please specify the advantages:
  3. Given your experience throughout the entire process was it worth the time and effort you put into the project: Yes/No. Please specify:
  4. Given your experience throughout the entire process would you do it again? Yes/No. Please specify.
  5. If you did it again/ had to do it again, would you change your approach and/ or procedure? Yes/No. If yes please specify:

**4. Any final topics and comments you would like to add:**

## **Developer Questions (Phase 2)**

**Section 1) General satisfaction**

- 1. On a whole are you satisfied with the developed XR application?

|  |  |  |  |  |
| --- | --- | --- | --- | --- |
| Extremely satisfied | very satisfied | Somewhat satisfied | not very satisfied | Not at all satisfied |

If no, please specify what aspects you are not satisfied with:

- 1. In your opinion, how likely is it that the developed XR application will be an effective and useful teaching tool for a university course?

|  |  |  |  |  |
| --- | --- | --- | --- | --- |
| Extremely likely | very likely | undecided | not very likely | no at all likely |

If you selected unlikely please specify why:

- 1. Based on your experience in content creation, please rate the level of difficulty of this XR application in terms of developing the tool:

|  | Very difficult | difficult | average | easy | Very easy |
| --- | --- | --- | --- | --- | --- |
| Subject matter/ complexity of content |  |  |  |  |  |
| Concept design and storyboarding |  |  |  |  |  |
| Graphic design (including UI, UX, menus) |  |  |  |  |  |
| Audio design |  |  |  |  |  |
| Coding |  |  |  |  |  |
| Data collection and reporting system |  |  |  |  |  |
| Bug fixing |  |  |  |  |  |
| Project Management |  |  |  |  |  |
| Communication with subject matter experts |  |  |  |  |  |
| overall |  |  |  |  |  |

- 1. As a hole, how would you rate the collaboration/ working relationship with the University of Newcastle?

|  |  |  |  |  |
| --- | --- | --- | --- | --- |
| Great working relationship | Good working | Neither good nor bad | Difficult working relationship | Extremely difficult working relationship |

If you indicated a difficult working relationship please specify why:

**Section 2) Feedback on developmental process**

- 1. How long did the developmental process take, absolute time start to finish?
  2. How many people were involved in the development of this application?
  3. How many working hours did the development of the tool take?
  4. Would you say the developmental process, as a whole, was an efficient straight forward process?

|  |  |  |  |  |
| --- | --- | --- | --- | --- |
| Extremely efficient and straight forward | very efficient | Somewhat efficient | Not efficient | Not at all efficient or straight forward |

If not please specify the factors contributing:

2.7 Where there any difficulties you did not anticipate whilst working on this application? Yes/No. If yes please specify:

- 1. Was working with UON staff members different from working with other commercial clients or subject matter experts from different areas? Yes/No. If yes please specify:

**3. Any final topics and comments you would like to add:**

## **Student pre-exposure/implementation survey (Phase 2)**

**Section 1) Exposure to VR/360 video or XR technologies.**

- 1. Did you have experience with VR/360 video (depending on respective application), either through personal or professional experience? *this question includes an information pop up explaining the term VR or 360 video* Yes/No
  2. How long have you spent directly engaged with VR/360 video? For example how long have you used a virtual reality headset or played around with the technology?

|  |  |  |  |  |
| --- | --- | --- | --- | --- |
| <5min | <1h | <10h | <100h | >100h |

- 1. How confident are you with the use of VR/360 video in a general context?

|  |  |  |  |  |
| --- | --- | --- | --- | --- |
| Extremely confident | very confident | Somewhat confident | not very confident | not at all confident |

- 1. Have you used simulation technology (either VR, AR or any other) in any of your classes or courses at the University of Newcastle? Yes/No.
  2. Before now, were you aware that you will have the opportunity to use an XR teaching tool in your course?

|  |  |  |
| --- | --- | --- |
| Aware | Somewhat aware | Not aware |

**Section 2) Expectations**

2.1 Using one word, how would you describe your MAIN response to the fact that you will be exposed to a VR/360 video training tool within this course?

|  |  |  |  |  |
| --- | --- | --- | --- | --- |
| Excited | curious | indifferent | nervous | terrified |

Other:

2.2 How confident are you that the XR teaching tool will be a helpful and effective teaching tool and help you reach your learning outcomes?

|  |  |  |  |  |
| --- | --- | --- | --- | --- |
| Extremely confident | very confident | Somewhat confident | not very confident | not at all confident |

2.3 Is there any reason that will personally prevent you from using a headset-based VR/360 video training tool? Yes/No/Maybe. If yes, specify:

**Section 3) Subject matter knowledge**

This section contains specific questions regarding the subject matter of each training tool. Questions were specific and designed to address existing knowledge and skill level of each learning objective. For each learning objective the following question types were asked:

3.1 Do you understand the underlying principles and theoretical background of the skill? Yes/No.

3.2 Have you ever practically applied the skill in a context appropriate manner? Yes/no.

3.3 How confident are you in applying the skill within its context]?

|  |  |  |  |  |
| --- | --- | --- | --- | --- |
| Extremely confident | very confident | Somewhat confident | not very confident | not at all confident |

## **Student post-exposure/implementation survey (Phase 3)**

**Section 1) Implementation feedback**

- 1. Did you have exposure to the headset-based training tool as part of your UoN course? Yes/NO

If no:

1.1.1 What was the reason for not using the tool?

- I missed the tutorial/ workshop at which it was presented
- There was not enough time for me to try it in the tutorial/ workshop
- VR makes me sick
- I did not want to put on the headset
- The headset did not fit/ was too uncomfortable to use
- Other, please specify……
  1. How long (answer in minutes) did you spend engaged with the teaching tool in total?
  2. In your opinion, was that enough time to explore the VR application and use the teaching tool?

|  |  |  |
| --- | --- | --- |
| Time spent with the tool was adequate | I would have liked more time | I would have liked a lot more time. |

1.4 Where there any technical problems that occurred during the use of the technology? Yes/No. If yes, please specify:

1.4 How satisfied were you with the way the teaching tool was integrated into your course?

|  |  |  |  |  |
| --- | --- | --- | --- | --- |
| Extremely satisfied | very satisfied | Somewhat satisfied | not very satisfied | not at all satisfied |

- 1. What did you like/dislike about the way it was integrated into your course?
  2. Would you prefer a different way of engaging with the VR teaching tool in the future? Yes/No. If yes, please specify:

**Section 2) General feedback**

2.1 How easy was the use and navigation within the XR tool?

|  |  |  |  |  |
| --- | --- | --- | --- | --- |
| Extremely easy | easy | Neither easy nor difficult | difficult | Very difficult |

2.2 How confident are you that you learned the content that was delivered via the VR tool?

|  |  |  |  |  |
| --- | --- | --- | --- | --- |
| Extremely confident | very confident | Somewhat confident | not very confident | not at all confident |

2.3 To what extent to you agree with the following statements:

2.3.1 I **enjoyed** using the VR teaching tool as part of the course

2.3.2 The VR tool provided an **additional** way of learning

2.3.3 I could not have **learned the content** in any other way

2.3.4 The tool **enhanced** my learning experience

2.3.5 The tool made the content **easier** to understand

2.3.6 The VR tool made it **unclear** what my learning goals were

2.3.7 I enjoyed the **practical** training aspect of the VR tool

2.3.8 Using the VR tool was a **waste of time**

2.3.9 I would have **preferred to use classical** teaching methods ONLY (such as textbooks and lecture or face to face tutoring)

2.3.10 The VR tool was cool but did not teach me anything new

2.3.11 The VR tool **bridges the gap** between textbook and practical training

|  |  |  |  |  |
| --- | --- | --- | --- | --- |
| strongly agree | agree | Neither agree nor disagree | disagree | strongly disagree |

2.4 In general what did you like/ dislike about the VR teaching tool?

2.7 In the future, would you like to use VR or any other type of XR technology in more of your courses at the university?

|  |  |  |
| --- | --- | --- |
| Yes | unsure | no |

If no, why not?

**Section 3) Subject matter knowledge**

This section contains specific questions regarding the subject matter of each training tool. Questions were designed to address knowledge and skill level after usage of the VR tool. For each learning objective the following question types were asked:

3.1 Do you understand the underlying principles and theoretical background of the skill? Yes/No.

3.2 Have you ever practically applied the skill in a context appropriate manner? Yes/no.

3.3 How confident are you in applying the skill within its context]?

|  |  |  |  |  |
| --- | --- | --- | --- | --- |
| Extremely confident | very confident | Somewhat confident | not very confident | not at all confident |
